# Experimental Validation and Multi-omics Analysis Identified ARPC1A as a Novel Oncogene and Potential Therapeutic Target in Glioblastoma

**DOI:** 10.7150/jca.94552

**Published:** 2024-05-30

**Authors:** Jun Yang, Chengcheng Xue, Zesong He, Li Ying, Wei Meng, Meihua Li, Na Zhang, Taohui Ouyang

**Affiliations:** 1 Department of Neurosurgery, the 1st affiliated hospital, Jiangxi Medical College, Nanchang University, No.17, Yongwai Street, Nanchang, Jiangxi province, 330006, China.; 2 Department of Neurology, the 1st affiliated hospital, Jiangxi Medical College, Nanchang University, No.17, Yongwai Street, Nanchang, Jiangxi province, 330006, China.

**Keywords:** ARPC1A, Tumor microenvironment, Glioblastoma, Proliferation, Temozolomide

## Abstract

Actin-related protein 2/3 complex subunit 1A (ARPC1A) is implicated in several cancers due to its critical role in regulating actin polymerization. However, the exact mechanism of ARPC1A in cancer remains unclear. This study aims to investigate the biological role of ARPC1A in various cancers and the regulatory role of ARPC1A in glioblastoma multiforme (GBM). We analyzed the expression differences, prognostic value, mutations, immune infiltration, immune microenvironment, and single-cell level correlations of ARPC1A in various cancers. Furthermore, we employed gene set enrichment analysis (GSEA) and functional experiments to elucidate the regulatory mechanisms of ARPC1A on GBM. Importantly, we assessed the role of ARPC1A in temozolomide (TMZ) resistance of GBM. ARPC1A expression was up-regulated in most cancer tissues and was associated with poorer prognosis. Genomic mutation analysis revealed that the predominant type of ARPC1A mutation in tumors was amplification. ARPC1A expression was negatively correlated with B-cell and immune scores in most tumors. Both GSEA and single-cell sequencing have revealed that ARPC1A promotes tumor proliferation and epithelial-mesenchymal transition. *In vitro* experiments confirmed that ARPC1A knockdown inhibited the proliferation and metastatic ability of GBM cells. Notably, silencing ARPC1A reduced TMZ resistance in GBM cells. This study highlights the prognostic value of ARPC1A in various tumors and its potential for application in immunotherapy. Meanwhile, the modulation of GBM malignant behavior and TMZ resistance by ARPC1A provides a new approach for personalized and precise treatment of GBM.

## Introduction

Cancer is a significant threat to human health worldwide in the 21st century, with one of the highest mortality rates 1. Despite advances in detection technologies, surgical and targeted therapies, the incidence continues to increase steadily 2. Meanwhile, the disability and mortality rates of cancer impose an economic burden on countries around the world 3. Therefore, it is crucial to identify a novel pan-cancer biomarkers to understand tumor genesis and adopt relevant immunotherapies. Pan-cancer research is an emerging approach to cancer research that can be combined with multi-omics to explore the mechanisms of tumor genesis 4.

The ARP2/3 complex is crucial for forming actin polymerization and cytoskeletal structure 5. The complex has been implicated in immune diseases, genetic disorders, and cancers 6-8. Aberrant expression of ARP2/3 can affect cancer cell proliferation, migration 9. ARPC1A is a subunit of the ARP2/3 complex and is essential in regulating actin polymerization. Silencing ARPC1A inhibits migration, invasion, and cytoskeleton formation of prostate cancer cells 10. Similar observations have been reported in studies of pancreatic cancer 11. High level of ARPC1A is a risk factor for patients with hepatocellular carcinoma 12. However, the role of ARPC1A has been mainly limited to individual tumor types, necessitating comprehensive studies encompassing pan-cancer datasets.

**Figure 1:**
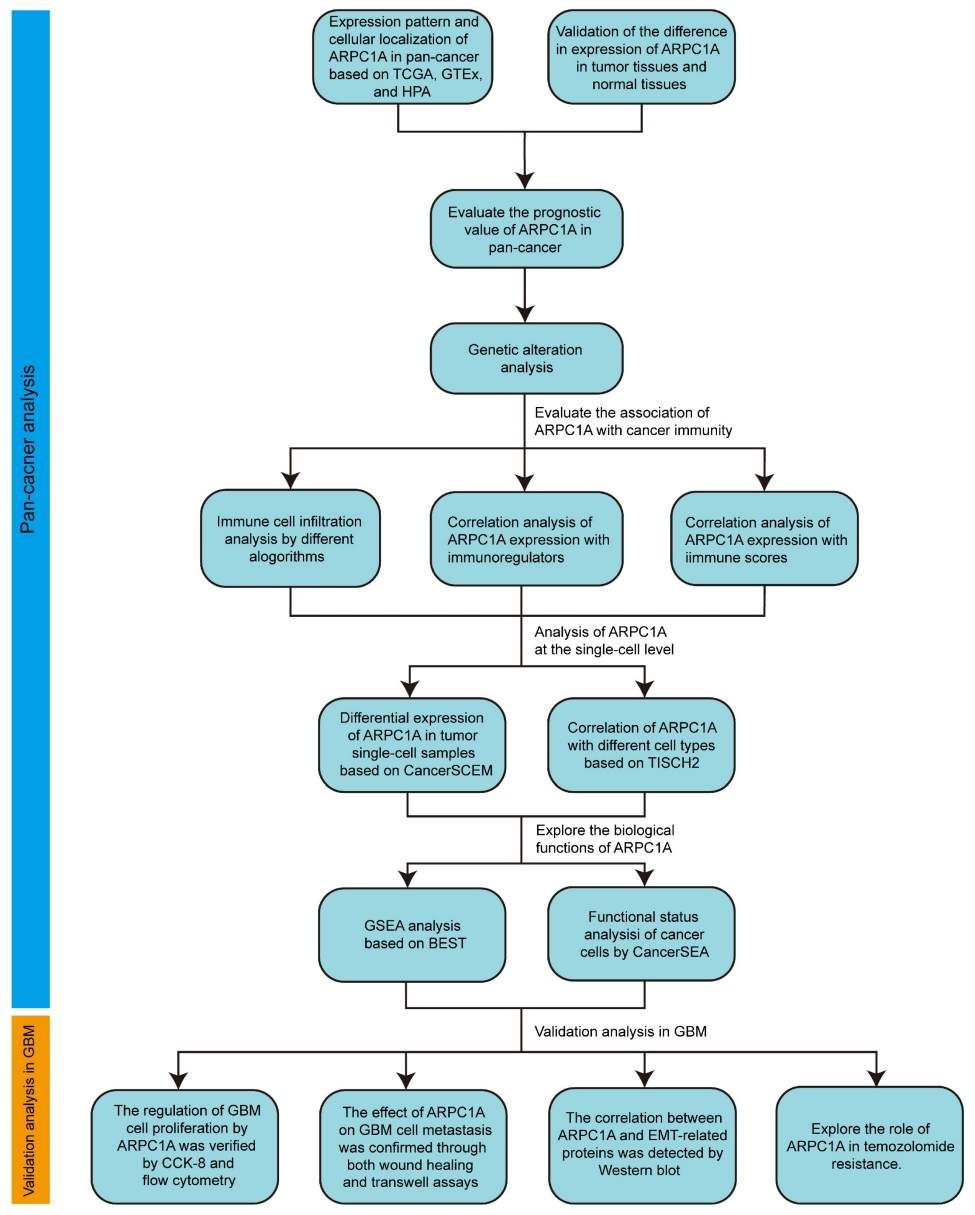
The flow chart of study.

In this study, we conducted a comprehensive pan-cancer analysis of ARPC1A utilizing multiple public databases and experimental validation. **Figure 1** illustrates the detailed research process. Initially, we identified the expression differences and prognostic value of ARPC1A across tumors. We investigated the correlation between ARPC1A and genomic mutations as well as immune microenvironments in various tumors. At the single-cell level, we examined the expression and biological functions of ARPC1A in different cell types. Additionally, we conducted experiments to determine the effects of ARPC1A on GBM cell proliferation, metastasis, and TMZ treatment. This research offers valuable insights into the role of ARPC1A in cancer development, particularly as a potential therapeutic target for GBM.

## Material and methods

### Collection of human samples

Four pairs of tumors and adjacent normal tissues were collected from patients who underwent surgical resection of gliomas at the First Affiliated Hospital of Nanchang University. The patients and the Ethics Committee agreed to collect and use the tissue specimens. To obtain tissue for glioma patients, we set inclusion and exclusion criteria. Inclusion criteria: (1) age >18 years; (2) patients with primary gliomas; (3) WHO grade IV gliomas (glioblastomas) only 13. Exclusion criteria:(1) Received chemotherapy or radiotherapy. The clinical parameters of all the patients in this study are displayed in supplementary Table S1.

### Data mining and expression differences

RNA sequencing data for 33 tumor tissues and 31 normal tissues were obtained from TCGA and GTEx (https://gtexportal.org/home/). Clinical data from the patients were obtained by downloading the harmonized and standardized datasets from the UCSC database. In the statistical process, we require that each group of samples be satisfied by more than three samples, and the variance of each group of samples should not be 0. The statistical analysis will only be performed if the conditions are satisfied. The ARPC1A expression data was analyzed using the Wilcoxon rank sum test and visualized using the “ggplot2” R package.

The HPA (http://www.proteinatlas.org/) provides comprehensive data on the distribution of proteins in human tissues and cells. Immunohistochemical images of ARPC1A were obtained from HPA. In addition, we extracted the subcellular localization of ARPC1A in cells and immunofluorescence images of A-431 (human epidermoid carcinoma cell line) and MCF-7 (human breast cancer cells) from HPA. Finally, we analyzed the downloaded data with the “ggplot2” R package to investigate the association between ARPC1A and the pathological stage of cancer.

### Prognostic analysis

The study analyzed four survival indicators: OS, DSS, DFI, and PFI. The Cox proportional hazards regression model was constructed with the “survival” R package to explore the prognostic association between ARPC1A expression and tumor prognosis. The Logrank test was applied for statistical significance testing. The expression threshold was set at 50% to distinguish high ARPC1A from low ARPC1A expression.

### Genomic mutation analysis

To assess the mutation characteristics of ARPC1A, we uesd the cBioPortal website (https://www.cbioportal.org/). We obtained information on ARPC1A mutations across 26 types of cancer using the “cancer types summary” module. Furthermore, we retrieved detailed information regarding the specific mutation sites within ARPC1A using the "mutation" module.

We downloaded the GDC (https://portal.gdc.cancer.gov/) for the genomic stability study. The TMB of the tumors was calculated using the “maftools” R package. The MSI scores from previous studies were extracted for MSI analysis 14. The above data was integrated with gene expression data, and log2(x+1) transformation was performed on all expression values. The final result was presented as a radar plot. Correlation data for MMR genes and ARPC1A were extracted from the TIMER database (http://timer.comp-genomics.org/) and visualized using the “ComplexHeatmap” R package.

### Tumor immune-infiltrating cells, immune scores, and immunoregulators analysis

The immune infiltration score was evaluated using three algorithms: QUANTISEQ, EPIC, and CIBERSORT, with the “IOBR” R package. Pearson correlation analysis was performed to determine the association between ARPC1A expression and the immune infiltration score. The stromal, immune, and estimate scores of each patient were evaluated using the “ESTIMATE” R package, and the results are presented as radar plots 15, 16. Finally, we extracted 60 immune-related genes from a previous study, including 24 inhibitory and 36 stimulatory genes 17. The "limma" R package evaluated the correlation between ARPC1A expression and immune-related genes.

### Single-cell analysis of ARPC1A

We interpreted the expression level of ARPC1A in single-cell tumor samples using the CancerSCEM single-cell database 18. Subsequently, we accessed TISCH2 to explore the expression of ARPC1A in different cell types. To further reveal the biological function of ARPC1A in cancer, we accessed the CancerSEA database 19. The functional status of ARPC1A in GBM and UM was obtained from the database, with a correlation coefficient threshold of 0.3 and a significance threshold of 0.05.

### GSEA

The BEST database (https://rookieutopia.com/app_direct/BEST/) is a platform that includes transcriptomic data for all cancers and is dedicated to the comprehensive exploration of cancer biomarkers. First, we selected 22 GBM datasets containing 2277 cases for analysis. Subsequently, the target genes were entered into the "single gene" module. We selected the enrichment analysis module in the interface and performed KEGG and Hallmark analyses. The results were presented in the form of a mountain ridge diagram. FDR less than 0.25 and corrected p-value less than 0.05 represent significant enrichment results.

### Cell culture and transfection

Four GBM cell lines (T98G, U87, U251, and U118) and one normal glial cell (SVG p12) were used in this study. T98G, U251, and U118 were generous donations from Haibin Wu, The First Affiliated Hospital of Nanchang University. U87 was obtained from Biowing Applied Biotechnology Co. (Shanghai, China). SVG p12 was obtained from Jenniobio Biotech Co. (Guangzhou, China). We maintained these cells under suitable conditions (37°C; 5% CO2). The siRNA sequences (Han Yi Biosciences, China) for ARPC1A knockdown were indicated in supplementary Table S2. We synthesized stably transfected shRNA and control lentivirus (Genechem, China) from siRNA sequences with optimal knockdown efficiency and negative controls. According to the manufacturer's instructions, the shRNAs or control viruses were co-cultured with cells for 72 h. Stable transfected cell lines were screened with 1 μg/ml puromycin.

TMZ-resistant cell lines (U87-TR, U251-TR) were constructed from parental cells (U87, U251). Specifically, the parental cell lines were cultured with progressively increasing concentrations of TMZ for six months to screen for TMZ-resistant GBM cell lines. U87-TR and U251-TR cell lines were maintained in daily culture with 50 μM TMZ (IT1330, Solarbio). All TMZ in the experiments was solubilized by dimethyl sulfoxide (D8371, Solarbio).

### Western blot

Protein samples were prepared by adding a lysis solution to the cells. Standard laboratory procedures were followed during the western blotting experiments. Specific information on antibodies was provided in supplementary Table S3.

### Wound-healing assay

Transfected cells were incubated in 6-well plates and scratched with a 200 μL pipette tip. Photographs were taken at the 0 and 24-hour time points, respectively.

### CCK-8 assay

The transfected cells were seeded into culture plates at 3 × 104 cells/mL per well, 100 µL. CCK-8 assays were performed at 0, 24, 48, and 72 h using a CCK-8 kit (AC11L054, Life-iLab). Absorbance was measured at 450 nm using a fully automated zymography system (Tecan, Switzerland).

### Cell invasion assay

Cell invasion assays were performed using transwell chambers (3422, Corning) equipped with a stromal gel (356234, Corning). Transfected cells were suspended in 200 µL of cell suspension and placed in the transwell chamber. After 24 h of incubation, cells were stained and fixed with 4% paraformaldehyde (P1110, Solarbio) and 0.5% crystalline violet solution (G1065, Solarbio). The cells were counted using an inverted microscope (Leica, Germany).

### Cell cycle assay

The transfected cells were collected, washed three times, and stained using the cycle kit (C543, DOJINDO LABORATORIES). The stained cells were placed at 30°C and 4 °C for 30 minutes, protected from light, respectively. The results were presented using flow cytometry at the end of incubation.

### Statistical analysis

All data calculations, data visualizations, statistical analyses, and bioinformatics analyses were performed using GraphPad Prism 9.0 software (GraphPad, USA) and R software version 4.2.2 (https://www.r-project.org/, accessed on January 2, 2023). We utilized Student's t-test to assess and compare the observed differences between the two groups. Differences between multiple groups were analyzed with ANOVA test. All experiments were conducted at least three times. The data are presented as mean ±SEM. Statistical significance was set at p < 0.05, which is considered statistically significant.

## Results

### Expression pattern of ARPC1A in human cancers

**Figure 2:**
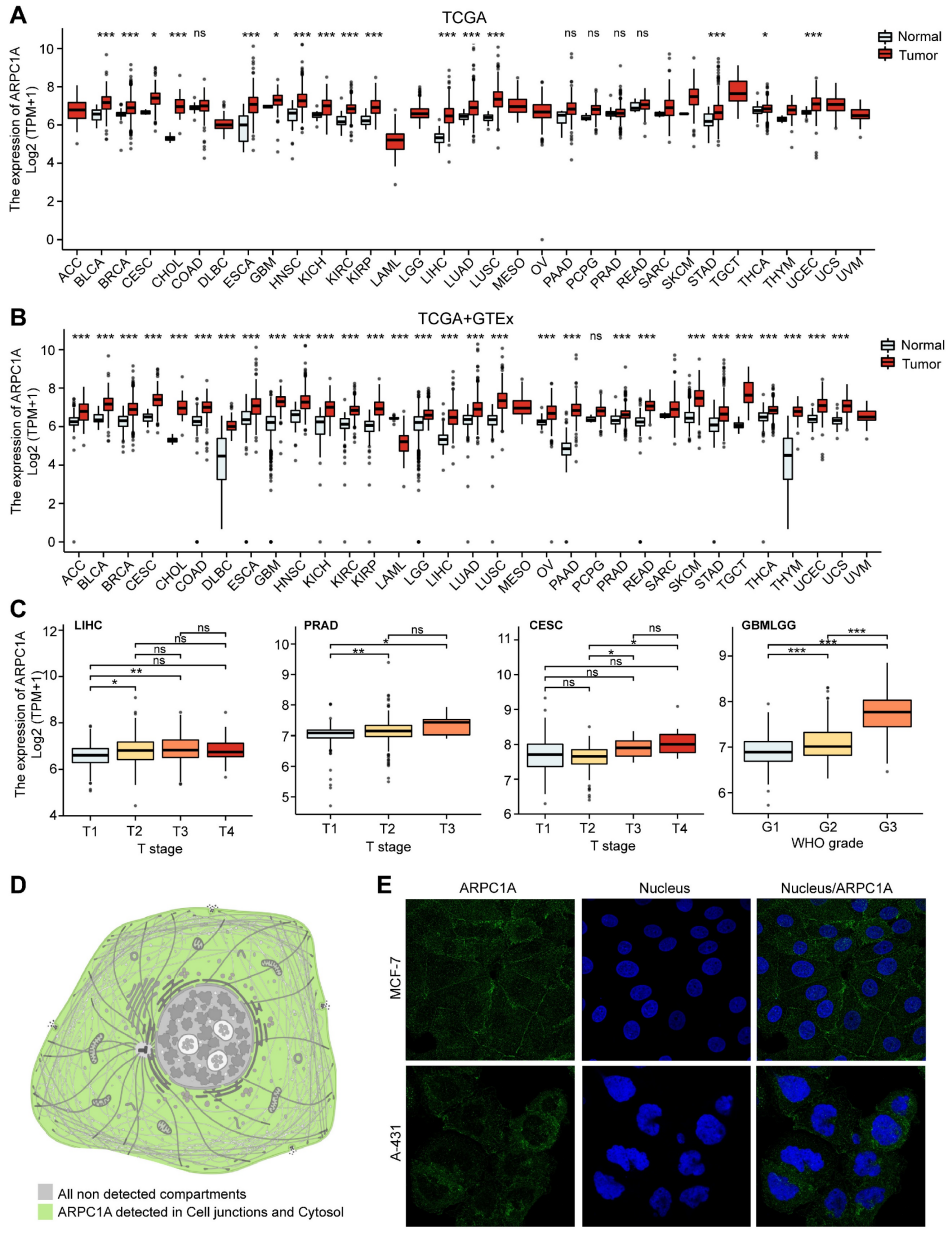
Expression pattern of ARPC1A in pan-cancer. **A** Difference in expression of ARPC1A in tumor and normal tissues based on TCGA database. **B** Difference in expression of ARPC1A in tumor and normal tissues based on TCGA and GTEx databases. **C** Correlation between ARPC1A expression and tumor stage. **D** Subcellular localization of ARPC1A in cells. **E** Immunofluorescence images of ARPC1A in MCF-7 and A-431 cell lines were obtained from the HPA database.

We initially analyzed the expression levels of ARPC1A in tumors and adjacent normal tissues. **Figure 2A** revealed that ARPC1A expression was up-regulated in 16 of 33 tumors. These tumors include BLCA, BRCA, CESC, CHOL, ESCA, GBM, HNSC, KICH, KIRC, KIRP, LIHC, LUAD, LUSC, STAD, THCA, and UCEC. Notably, low expression of ARPC1A was not observed in these tumors.

Since some tumors' normal tissue data was unavailable in the TCGA database, we obtained normal tissue data from GTEx and performed an integrated analysis. **Figure 2B** illustrated that apart from the high expression observed in TCGA, ARPC1A displayed up-regulated in ACC, COAD, DLBC, PAAD, PRAD, LGG, READ, SKCM, TGCT, OV, THYM, and UCS. Conversely, ARPC1A exhibited downregulation in LMAL (**Figure 2B**). We also downloaded 12 paired tumor samples from TCGA for validation (**Figure S1**). To examine the function of ARPC1A in tumor progression, we explored the expression of ARPC1A in different stages of tumors. We found that ARPC1A expression was positively correlated with the stages of LIHC, PRAD, CESC, GBM/LGG, KICH, PAAD, KIRP, and KIRC, except for COAD (**Figures 2C and S2A**). At the cellular level, we observed that ARPC1A was predominantly localized in the cell junctions (**Figure 2D**). Subsequently, we determined that ARPC1A was predominantly present in the cell junctions and cytoplasm by immunofluorescence in A-431 (human epidermoid carcinoma cell line) and MCF-7 (human breast cancer cells) (**Figures 2E and S2B**). This localization is consistent with ARPC1A playing an essential role in cytoskeleton formation.

### Differential expression of ARPC1A protein in cancers

**Figure 3:**
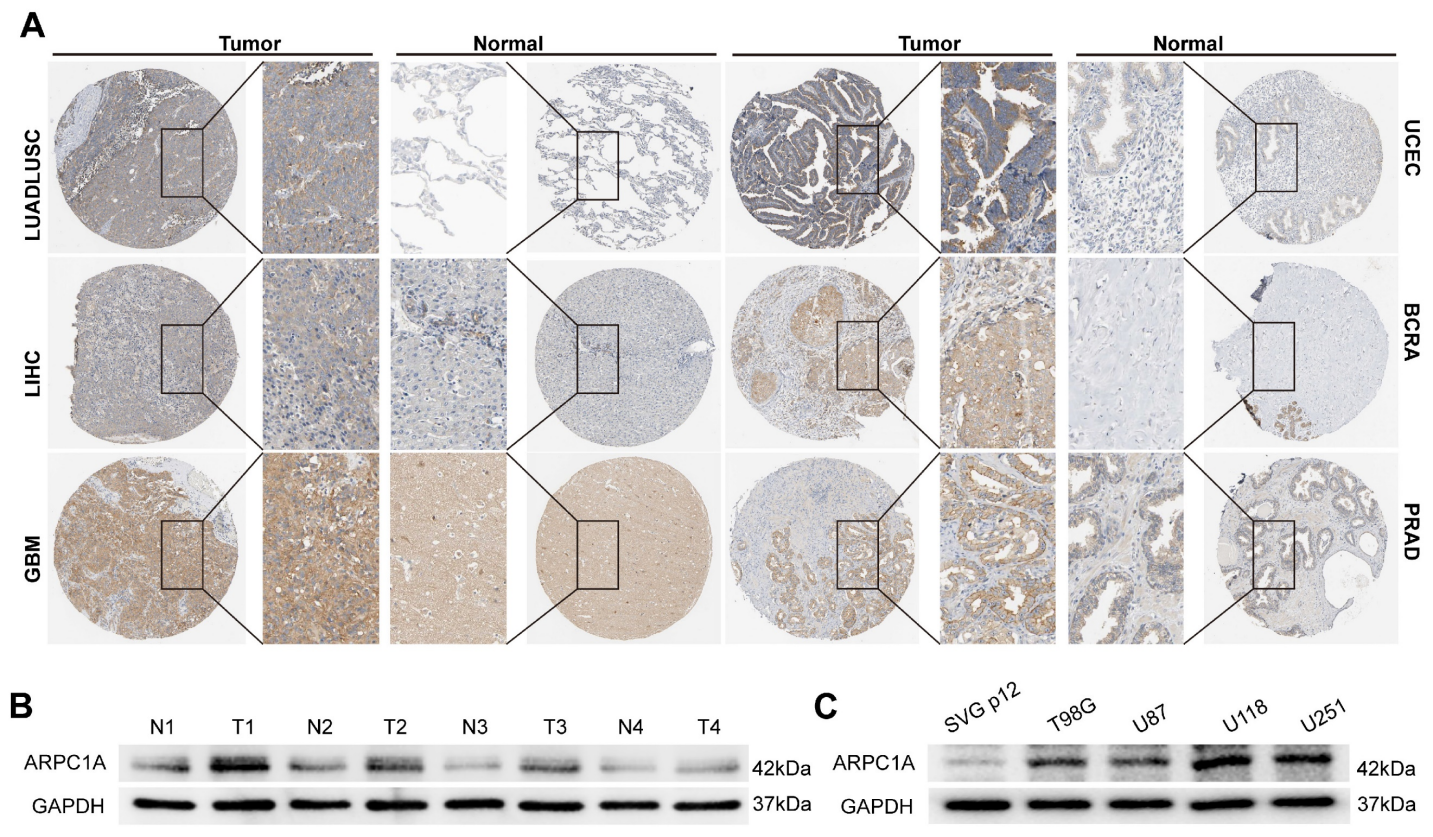
Differential expression of ARPC1A protein in various tumors. **A** Immunohistochemical images of ARPC1A in six pairs of tumors and normal tissues. **B** Western blot detection of ARPC1A expression in 4 pairs of gliomas and adjacent normal tissues. N, non-tumor; T, tumor**. C** Western blot detection of ARPC1A expression in GBM cells (U87, U251, T98G, U118) and glial cells (SVG p12).

Protein expression in different cells often determines their specific function and state. We acquired six pairs of immunohistochemical images of tumors and normal tissues from the HPA database (**Figure 3A**). Interestingly, ARPC1A protein levels were consistent with mRNA levels in tumors. Therefore, we performed experimental validation in GBM tissues and cell lines. The results revealed that the expression of ARPC1A was higher in GBM tissues than in normal tissues (**Figure 3B**). Similarly, ARPC1A expressions were higher in GBM cell lines than in normal glial cells (**Figure 3C**). The different expression patterns of ARPC1A in various cancers suggest that ARPC1A may play specific functional roles in different tumors. Understanding the mechanistic underpinnings of these differences can provide insights into the molecular mechanisms of cancer and may provide directions for subsequent targeted therapies.

### Prognostic value of ARPC1A in cancer

**Figure 4:**
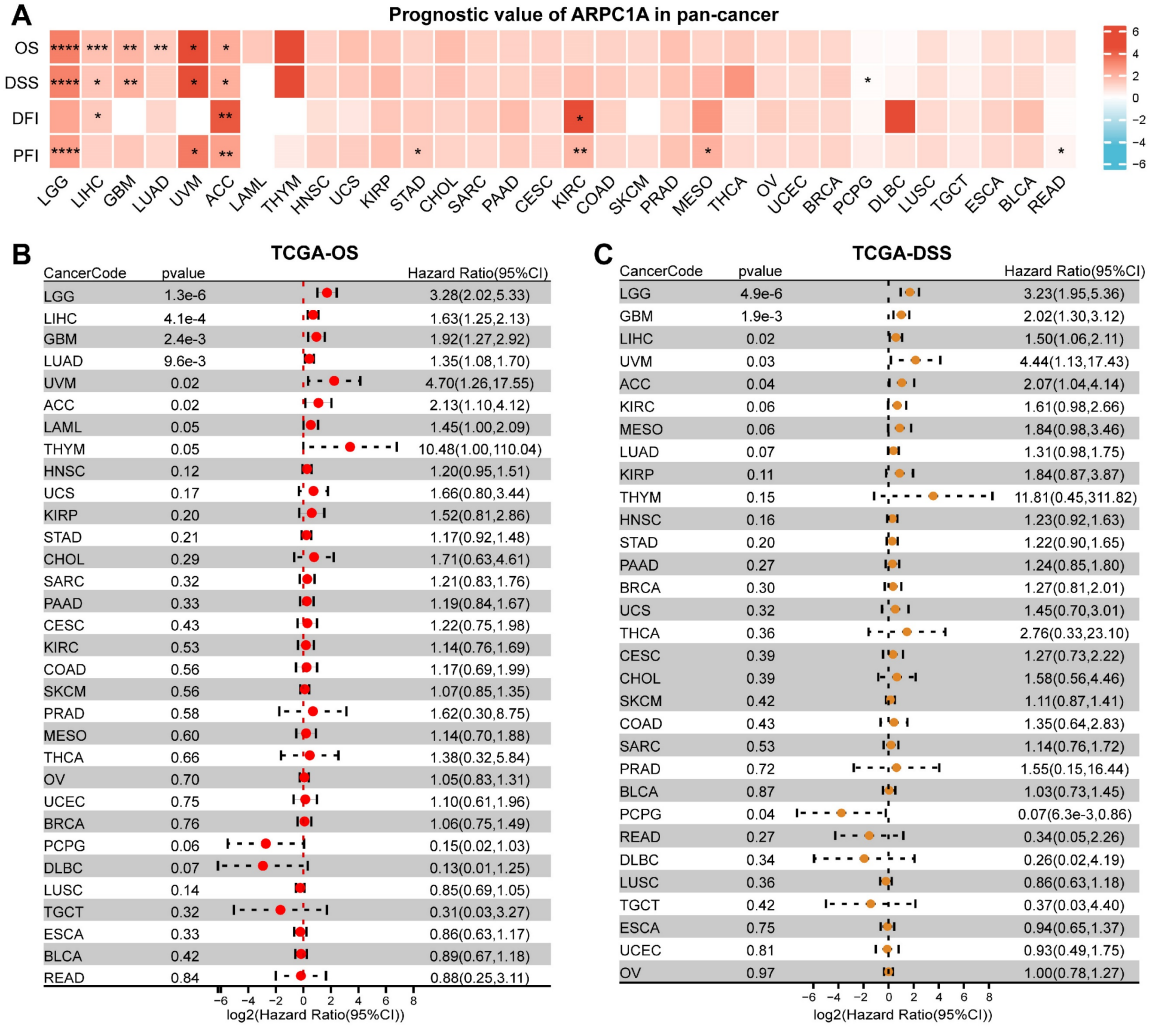
Prognostic value of ARPC1A in various tumors. **A** Correlation of ARPC1A expression with survival indices in various tumors, including overall survival (OS), disease-specific survival (DSS), disease-free interval (DFI), and progression-free interval (PFI). **B-C** The forest plot showed the correlation of ARPC1A with OS and DSS in tumors using the univariate Cox regression.

To evaluate the correlation between ARPC1A mRNA levels and tumor prognosis, we downloaded OS, DSS, DFI, and PFI data of patients with different tumors. The heatmap revealed that the expression of ARPC1A was positively correlated with the prognostic indicators of multiple tumors, except for READ and PCPG (**Figure 4A**). Specifically, the forest plot revealed that higher ARPC1A expression was associated with poorer OS in LGG, LIHC, GBM, LUAD, UVM, and ACC patients (**Figure 4B**). Similarly, higher ARPC1A expression in DSS analysis predicted poorer prognosis in LGG, LIHC, GBM, LUAD, UVM, and ACC patients and protected against PCPG (**Figure 4C**). DFI analysis indicated high ARPC1A expression was a risk factor for ACC, KIRC, and LIHC patients (**Figure S3A**). Moreover, higher ARPC1A was associated with poorer PFI in LGG, UVM, ACC, STAD, KIRC, and MESO patients, while the opposite effect was seen in READ patients (**Figure S3B**).

### Genomic mutation analysis of ARPC1A in cancer

**Figure 5:**
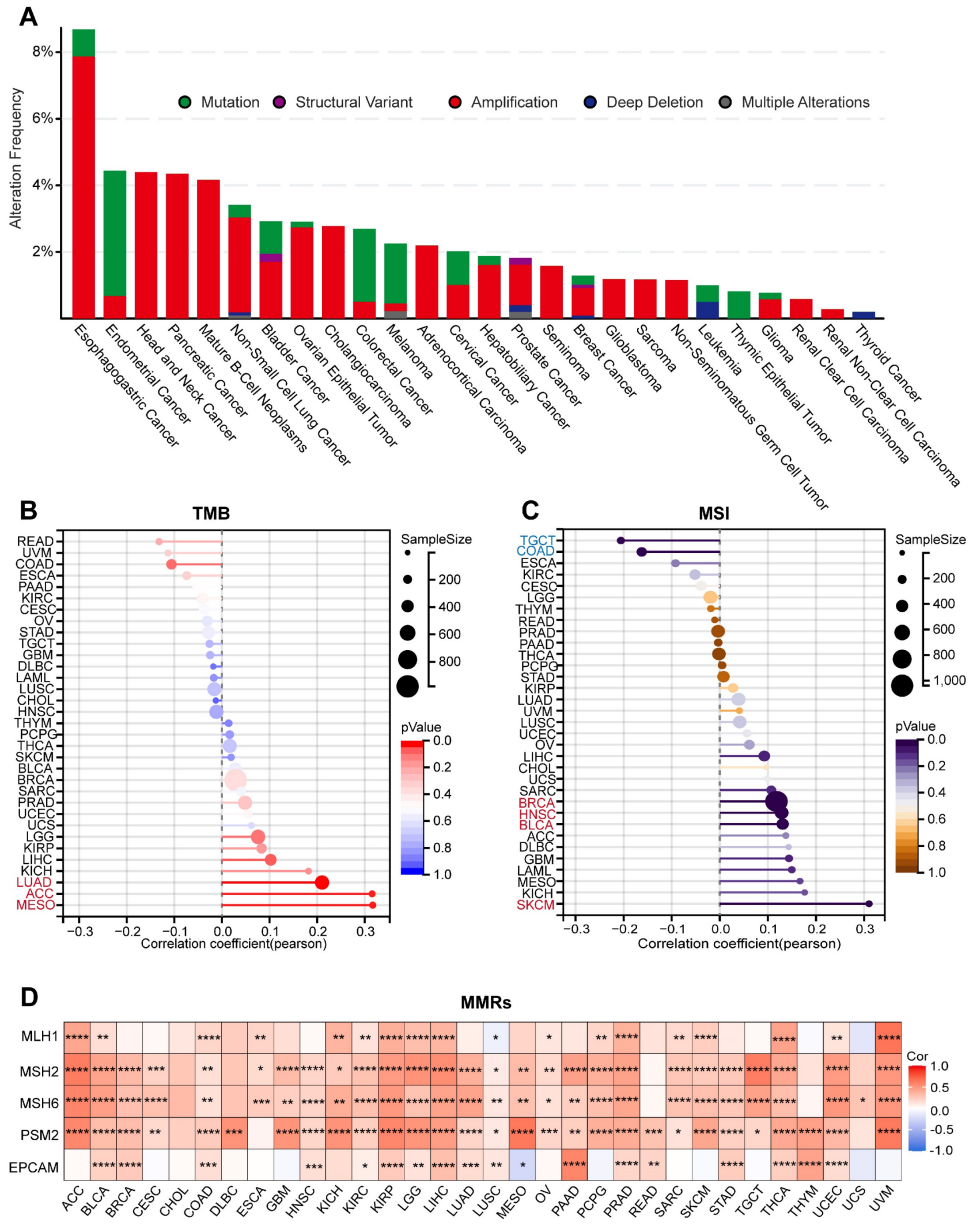
Genomic mutation analysis of ARPC1A in various tumors. **A** Mutation types and variant frequencies of ARPC1A in pan-cancer. **B** The lollipop graph displayed the correlation of ARPC1A expression in different cancers with TMB and MSI. **C** The heatmap revealed the correlation of ARPC1A expression with five key genes in MMRs, including MLH1, MSH2, MSH6, PSM2, and EPCAM.

Defects in the function of genome-stabilizing factors cause genomic instability and mutations, accumulating mutations in oncogenes. To reveal the genomic mutations of ARPC1A in cancer, we analyzed the mutation frequency of ARPC1A in tumors. The results indicated that ARPC1A had the highest mutation frequency in UCEC, COAD, and SKCM (**Figure S4**). Subsequently, we explored the different statuses of ARPC1A gene mutations. The results showed that amplification was the primary type of change. Specifically, the amplification rate of ARPC1A in esophagogastric cancer was the highest (>6%) (**Figure 5A**). TMB, MSI, and genetic instability were associated, and abnormalities in these metrics tend to increase tumor cell evasion of immunotherapy and resistance to chemotherapeutic agents 20, 21. **Figure 5B** revealed that ARPC1A expression was positively correlated with TMB in LUAD, ACC, and MESO. Moreover, ARPC1A expression was positively correlated with MSI in BRCA, HNSC, BLCA, and SKCM while negatively correlated in TGCT and COAD (**Figure 5C**).

The MMR genes accurately recognizes and repairs base mismatches generated during DNA replication or recombination, which is essential for maintaining genetic stability. We evaluated the correlation of five MMR genes (MLH1, MSH2, MSH6, PSM2, and EPCAM) with ARPC1A. The results showed that, except for CHOL, DLBC, READ, THYM, and UCS, ARPC1A was closely associated with MMR genes in the remaining 28 tumors (**Figure 5D**).

### Correlation of ARPC1A with TME in cancer

**Figure 6:**
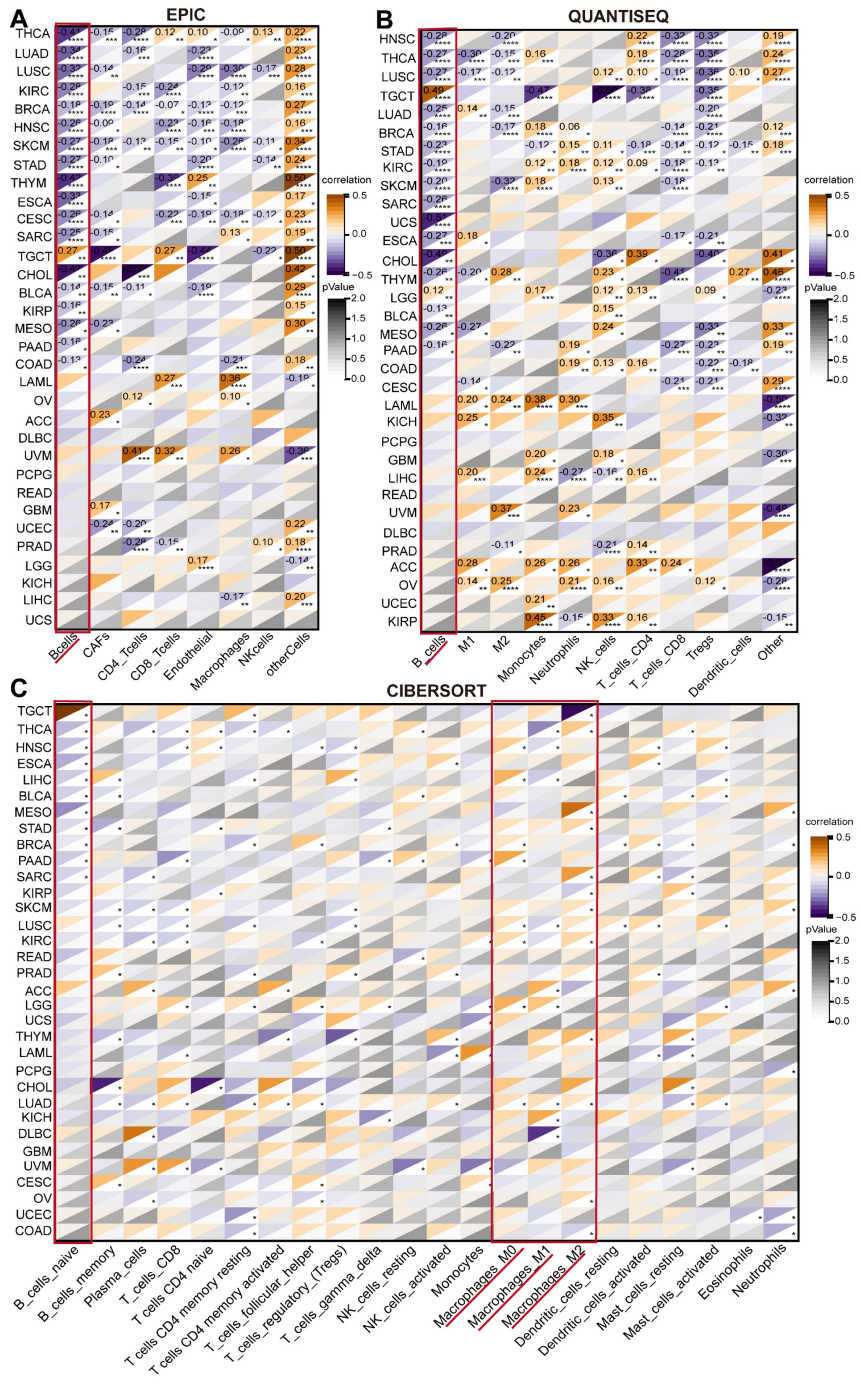
Correlation of ARPC1A expression with immune infiltrating cells in various cancers. **A-C** The correlation of ARPC1A expression with immune cell levels was evaluated based on three algorithms, QUANTISEQ, EPIC, and CIBERSORT.

Tumor cells typically colonize normal tissues and form the tumor microenvironment with stromal cells, immune cells, vascular endothelial cells, and extracellular matrix 22. Immune cells have a dual role in the TME, both promoting and inhibiting tumor immunity. Therefore, we explored the relationship between ARPC1A expression and immune-related factors. We calculated various immune cell infiltration scores based on several well-recognized algorithms (QUANTISEQ, EPIC, and CIBERSORT) to evaluate the association between ARPC1A levels and immune cell levels (**Figure 6A-C**). Interestingly, we observed that ARPC1A expression in the EPIC algorithm was negatively correlated with B cells of most cancers (**Figure 6A**). This phenomenon was confirmed in the QUANTISEQ algorithm (**Figure 6B**). To further validate the correlation between more immune cells and ARPC1A, we used the CIBERSORT algorithm. The results revealed that ARPC1A was positively correlated with M0-, M1-, and M2-type macrophages in most cancers, whereas it was negatively correlated with naive B cells (**Figure 6C**).

**Figure 7:**
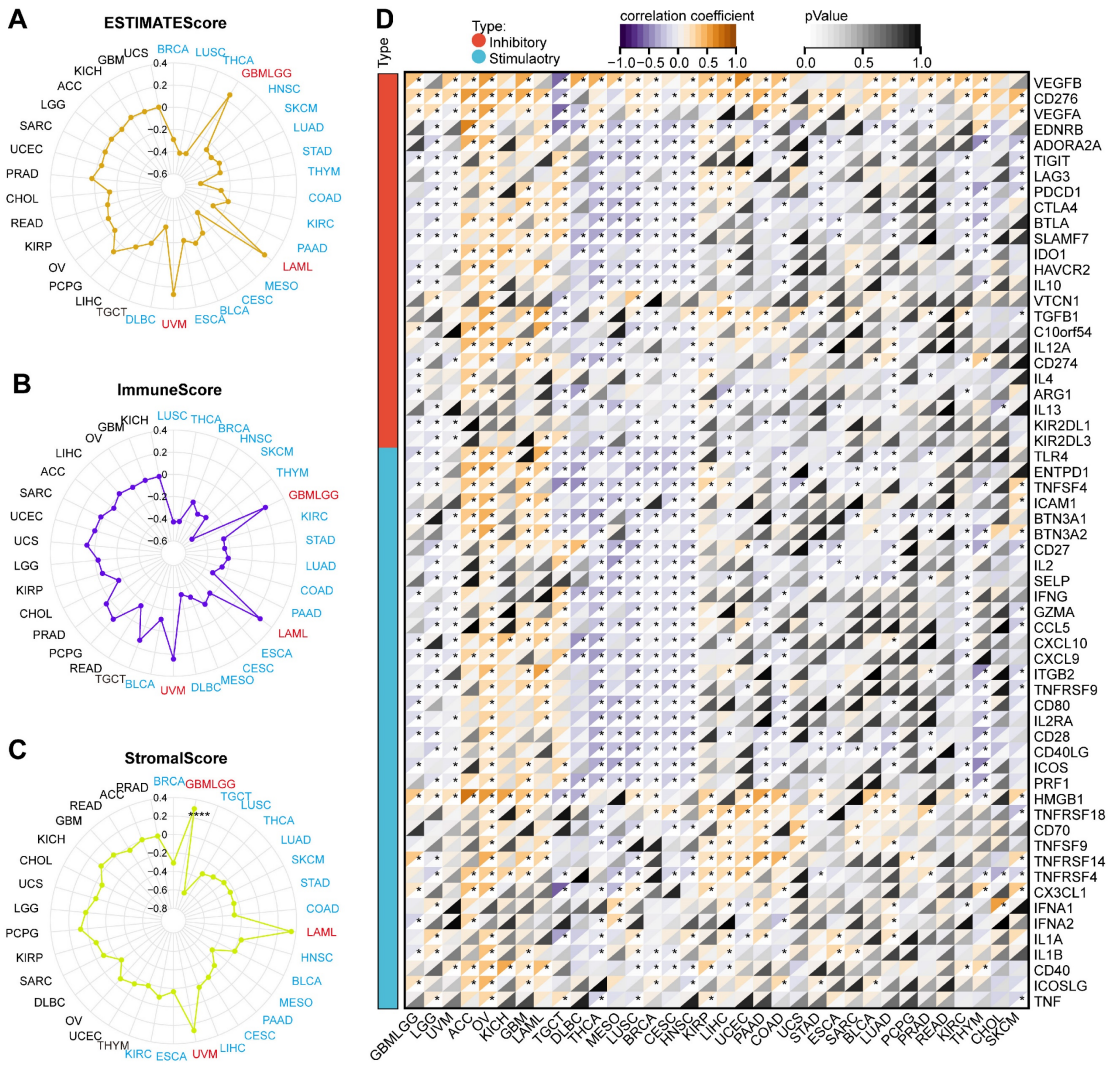
Association of ARPC1A expression with immune scores and immunoregulators in various cancers. **A-C** Correlation assessment of ARPC1A expression with estimated score, immune score, stromal score in pan-cancer. **D** Correlation of ARPC1A expression with immune inhibitory and stimulatory genes analyzed.

The ESTIMATE algorithm could evaluate the tumor microenvironment's composition, structure, and status, which was vital in studying tumor biological characteristics and prognosis and screening targeted drugs. We employed three scoring methods based on the ESTIMATE algorithm. ARPC1A expression was negatively correlated with the estimated score in 16 tumors including BRCA, LUSC, THCA, HNSC, SKCM, LUAD, STAD, THYM, COAD, KIRC, PAAD, MESO, CESC, BLCA, ESCA, and DLBC, while was positively correlated with estimated scores in GBMLGG, LAML and UVM (**Figure 7A**). The correlation between ARPC1A and immune scores was consistent with the results of the estimated score (**Figure 7B**). According to stromal scores, ARPC1A expression was negatively correlated with stromal scores in BRCA, TGCT, LUSC, THCA, LUAD, SKCM, STAD, COAD, HNSC, BLCA, MESO, PAAD, CESC, LIHC, ESCA, and KIRC, and positively correlated with GBMLGG, LAML, and UVM (**Figure 7C**). Moreover, we observed the correlation between ARPC1A expression and immune checkpoints. Genetically, ARPC1A was negatively correlated with almost all immune checkpoints in THCA, MESO, LUSC, BRCA, CESC, and HNSC. From a pan-cancer perspective, ARPC1A expression was positively correlated with VEGFB and CD276 (**Figure 7D**).

### Analysis of ARPC1A at the single-cell level

**Figure 8:**
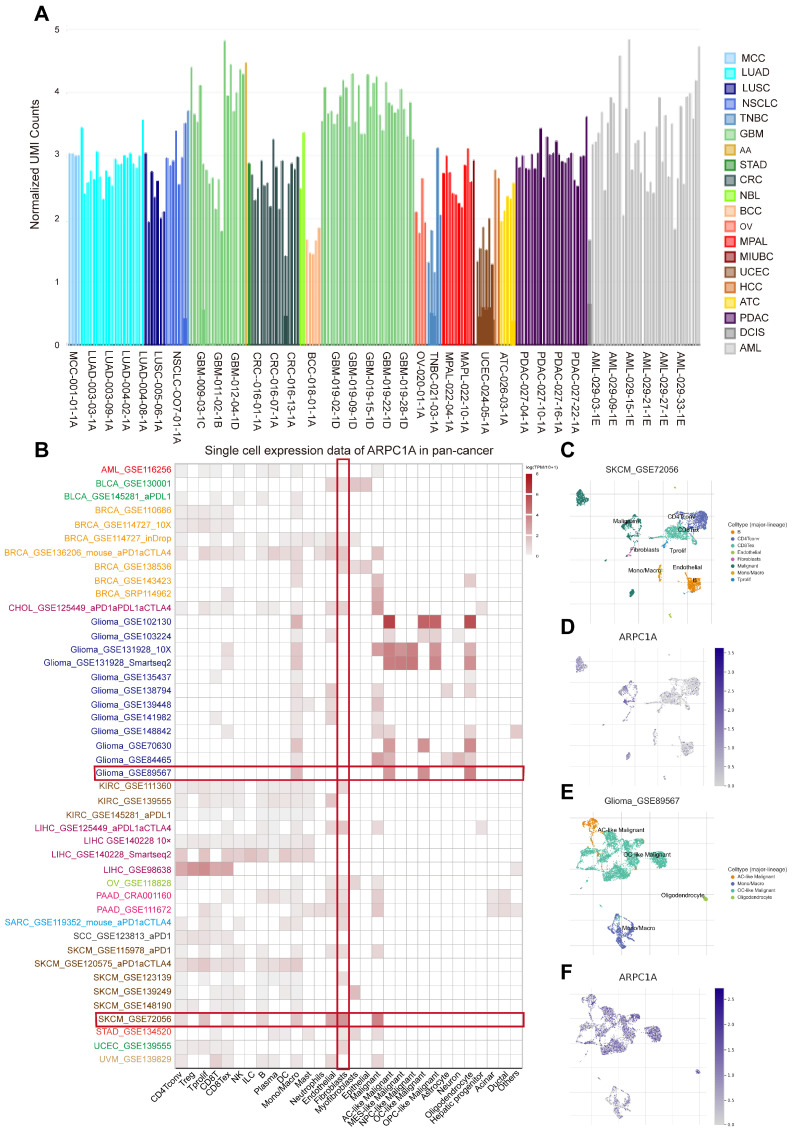
Single-cell sequencing analysis of ARPC1A in tumor samples. **A** Expression levels of ARPC1A in various tumor single-cell samples. **B** Correlation of ARPC1A expression in 15 tumor samples with 30 cell types. **C-F** Umap plots revealed the clustering of different cell types and expression levels of ARPC1A in GBM and SKCM tissues.

At the single-cell level, we analyzed the expression of ARPC1A in tumor single-cell samples. **Figure 8A** indicated that the expression of ARPC1A was higher in OV, UCEC, and TNBC than in other tumor tissues. Subsequently, we quantified the expression levels of ARPC1A in 30 cell types utilizing the TISCH2 database. The heatmap revealed that ARPC1A was widely expressed in various immune, functional, and malignant cells (**Figure 8B**). We observed that ARPC1A was predominantly expressed in B, CD4T, CD8T, endothelial, fibroblasts, tprolif, malignant, and marco cells of SKCM (**Figure 8C, D**). In GBM (GBM_GSE89567), ARPC1A was predominantly expressed in malignant, oligodendrocyte, and macro cells but not in fibroblasts (**Figure 8E, F**). Notably, this differential expression pattern was also seen in other tumors, suggesting a potential regulatory role for ARPC1A in the function of fibroblasts.

**Figure 9:**
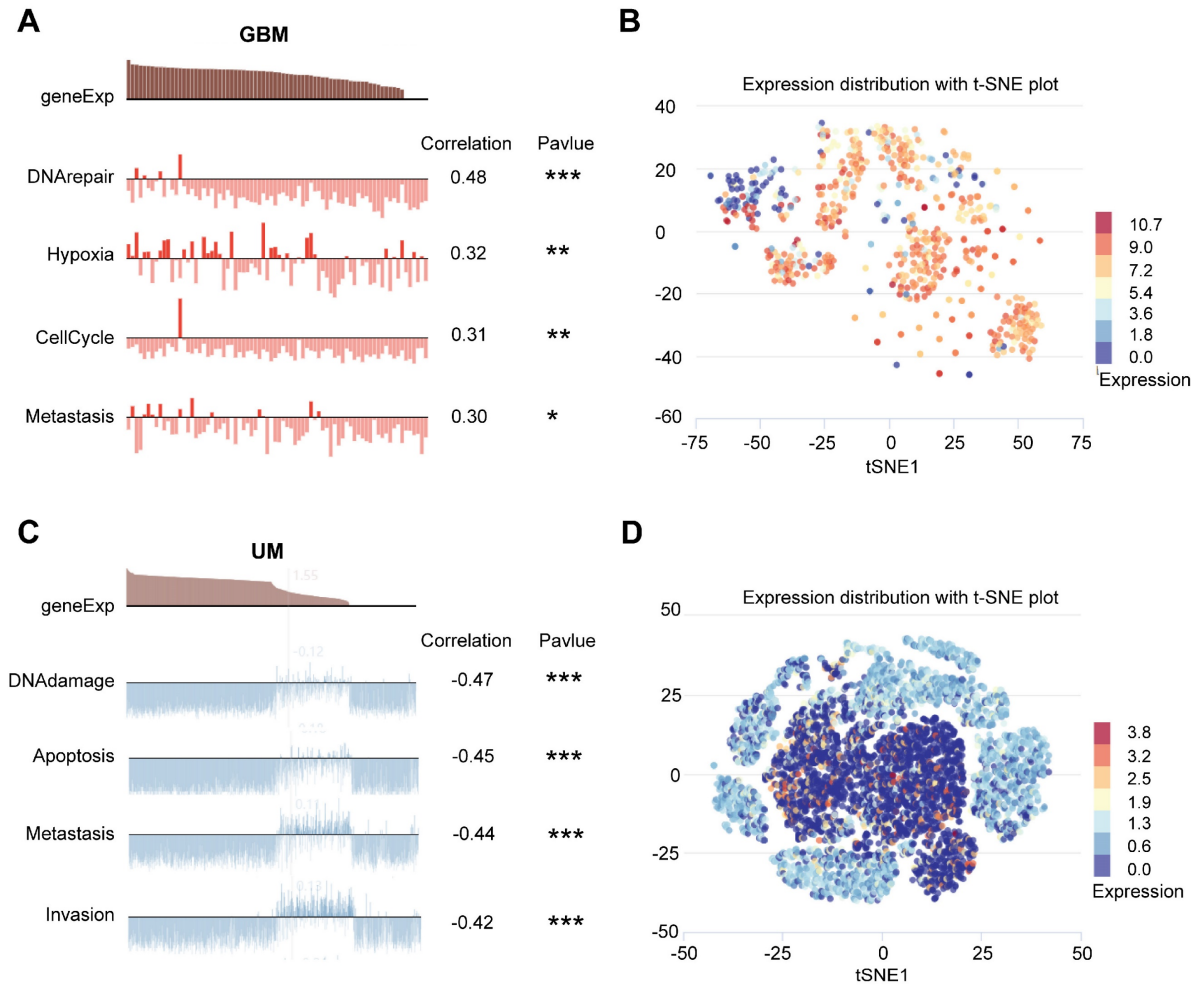
Single-cell sequencing analyses showed that ARPC1A was associated with biological functions in different cancers. **A** GBM showed that ARPC1A was positively associated with DNA repair, hypoxia, cell cycle, and metastasis. **B** The t-SNE plot showed the distribution of ARPC1A expression in GBM samples. **C** UM showed that ARPC1A was negatively correlated with DNA damage, apoptosis, metastasis, and invasion. **D** The t-SNE plot showed the distribution of ARPC1A expression in UM samples.

We then explored the functional status of ARPC1A at the single-cell level. CancerSEA showed that ARPC1A was positively correlated with DNA repair, hypoxia, cell cycle, and metastasis in GBM (**Figure 9A, B**). However, ARPC1A may play an inhibitory role in UM. **Figures 9 C and D** showed that ARPC1A negatively correlates with DNA damage, apoptosis, metastasis, and invasion in UM.

### Silencing ARPC1A inhibits proliferation and metastasis of GBM cells

**Figure 10:**
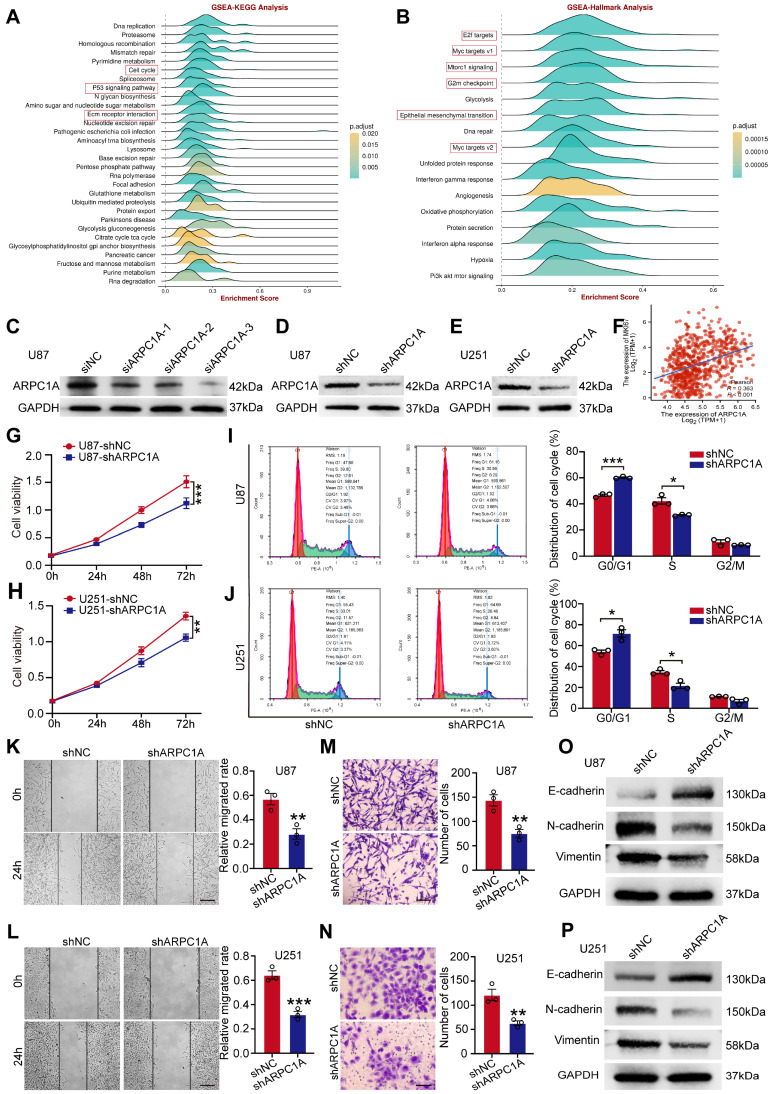
ARPC1A knockdown inhibited the malignant behavior of GBM cells. **A-B** GSEA analysis in 22 GBM datasets showed that ARPC1A was associated with critical genes for EMT and proliferation. **C** Western blot detection of ARPC1A expression after siRNA interference. **D-E** Western blot assay for knockdown efficiency in stably transfected cell lines. **F** Scatter plots showed that ARPC1A was positively correlated with Ki-67, an essential gene for proliferation. **G-J** CCK-8 assay with flow cytometry showed that ARPC1A knockdown inhibited the proliferative viability of GBM cells. **K-N** Wound healing assay and transwell assay showed that ARPC1A knockdown inhibited the migration and invasion of GBM cells. Scale bar:200μm (K-L), 100μm (M-N) **O-P** Western blot detection of the expression of epithelial cell marker E-cadherin and mesenchymal cell markers N-cadherin and vimentin after ARPC1A knockdown.

Previous studies have revealed that ARPC1A expression was up-regulated in GBM and was associated with poor prognosis. We aimed to investigate the functional role of ARPC1A in the pathogenesis of GBM. KEGG indicated that ARPC1A expression was positively correlated with various malignancy signaling pathways such as cell cycle, p53 signaling pathway, and ECM-receptor interaction (**Figure 10A**). Hallmark analysis indicated that ARPC1A expression was positively correlated with cell proliferation signaling pathways (E2F, MYC, mTORC1, G2M) and EMT (**Figure 10B**). These results suggested that ARPC1A may be a potential oncogene in GBM.

Finally, we selected U87 and U251 cell lines to validate the oncogenicity of ARPC1A *in vitro*. We interfered with the expression of ARPC1A by siRNA (**Figure 10C**). The siRNA sequences with the highest interference efficiency were selected to synthesize lentivirus and transfected into U87 and U251 cells. **Figures 10D** and** E** showed that ARPC1A was significantly down-regulated in both cell lines. Scatter plots indicated that ARPC1A was positively correlated with the proliferation of the essential gene Ki-67 in GBM (**Figure 10F**). ARPC1A knockdown inhibited the proliferation rate of U87 and U251 compared to controls (**Figure 10G, H**). Furthermore, flow cytometry revealed that ARPC1A knockdown resulted in cell cycle arrest at the G1 phase (**Figure 10I, J**). After that, we investigated the effect of ARPC1A on GBM metastasis. We found that ARPC1A knockdown significantly inhibited the migration and invasion ability of U87 and U251 compared with the controls (**Figure 10K-N**). EMT validation indicated that E-cadherin was significantly up-regulated after silencing ARPC1A, while N-cadherin and vimentin showed the opposite trend (**Figure 10O, P**). This result also coincided with the Hallmark analysis (**Figure 10B**). In conclusion, these findings emphasize that ARPC1A plays a critical role in developing GBM.

### Silencing ARPC1A promoted sensitization of GBM cells to TMZ

**Figure 11:**
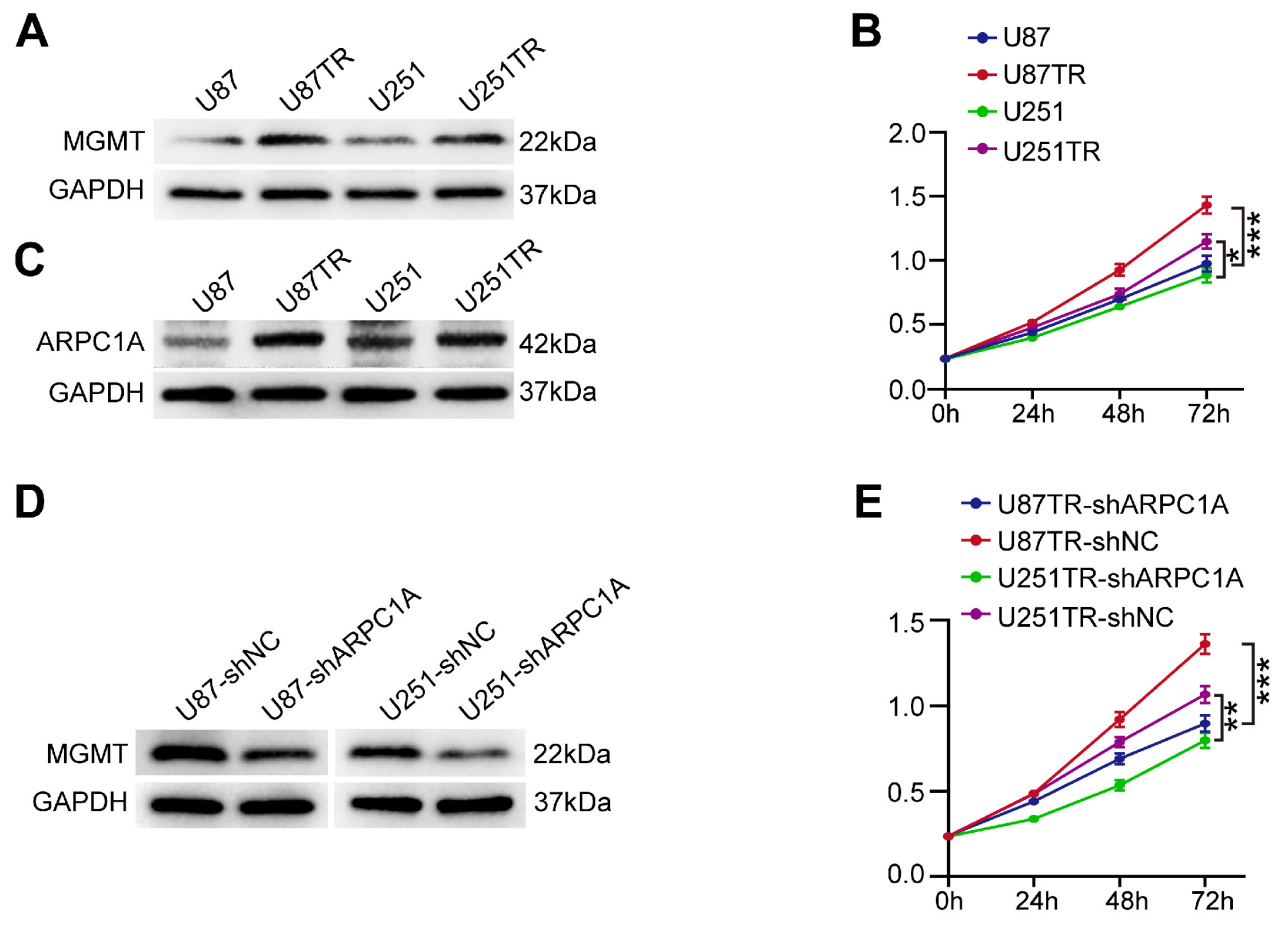
ARPC1A knockdown increases the sensitivity of GBM cells to TMZ. **A** Western blot was performed to detect the expression of MGMT in parental cells (U87, U251) and TMZ-resistant cell lines (U87TR, U251TR). **B** CCK-8 assay revealed higher cell viability of U87TR and U251TR than U87 vs. U251 at 50 μg/ml TMZ culture. **C** Western blot was performed to detect the expression of ARPC1A in parental cells (U87, U251) and TMZ-resistant cell lines (U87TR, U251TR). **D** Western blot detection of MGMT expression levels in TMZ-resistant cells stably transfected with ARPC1A knockdown. **E** CCK-8 assay showed that the cell viability of TMZ-resistant cell lines was lower than that of transfected controls after stable transfection with ARPC1A knockdown under 50 μg/ml TMZ culture conditions.

TMZ is an alkylating agent and is currently the first-line chemotherapeutic agent for treating GBM 23. We experimentally validated two TMZ-resistant cell lines (U87TR and U251TR) that were constructed for six months. The results indicated that MGMT was expressed at higher levels in the resistant cells than in the parental cells (**Figure 11A**). MGMT could directly repair the damage to oncogenes caused by TMZ, thereby inhibiting the killing of tumor cells by TMZ alkylating agents 24. Furthermore, the proliferation ability of U87TR and U251TR cells was higher than that of the parental cells when cultured with 50 μg/ml TMZ (**Figure 11B**). Therefore, we investigated the role of ARPC1A in treating GBM with TMZ. As shown in **Figure 11C**, the expression level of ARPC1A was higher in U87TR and U251TR cells than in parental cells. Subsequently, we established stable knockdown cell lines in TMZ-resistant cells. **Figure 11D** revealed that MGMT expression significantly decreased in ARPC1A knockdown compared to control. Additionally, the CCK-8 assay was used to assess the role of ARPC1A in the proliferation of TMZ-resistant cells, which demonstrated that ARPC1A knockdown inhibited cell proliferation (**Figure 11E**). These results suggest that ARPC1A knockdown increases the sensitivity of GBM to TMZ treatment.

## Discussion

ARPC1A is a gene that constructs a critical component of the cytoskeleton. The encoded protein is crucial in cytoskeletal reassembly and cell membrane remodeling. It plays an essential function in physiological processes such as cell movement, maintenance of cell shape, and cell division, which is also critical for the metastasis and spread of tumor cells. Previous studies reported that ARPC1A expression was upregulated in prostate cancer, and overexpression promoted lung metastasis of tumor cells 10. In addition, silencing ARPC1A inhibited the malignant behavior of prostate cancer cells in a ferroptosis manner 25. In hepatocellular carcinoma, ARPC1A was shown to be highly expressed and associated with poor prognosis 12. However, the exact mechanism by which ARPC1A affects tumor development remains unknown.

To the best of our knowledge, this is the first comprehensive investigation of the mechanism of ARPC1A in tumor development. Furthermore, we innovatively found that ARPC1A acts as an oncogene in GBM and can influence TMZ resistance. The study found that ARPC1A expression was up-regulated in all tumors examined, which was also verified in GBM tissues and cell lines. These results are consistent with previous studies. Moreover, ARPC1A expression was positively correlated with pathological stages of LIHC, PRAD, CESC, GBM/LGG, KICH, PAAD, KIRP, and KIRC. These correlations are crucial in predicting the oncogenicity of ARPC1A in tumors. In single-cell tumor samples, ARPC1A expression was higher in OV, UCEC, and TNBC compared to other tumors. This finding highlights that differential expression of ARPC1A may contribute differently to the development of various tumors. In assessing prognostic value, it was observed that high expression of ARPC1A was positively associated with poor prognosis in LGG, LIHC, GBM, LUAD, UVM, ACC, KIRC, STAD, and MESO. Overall, we explored the expression and prognostic value of ARPC1A in pan-cancer and GBM from multiple perspectives, highlighting its great potential for application in clinical diagnosis.

The abnormal and uncontrolled growth of normal cells, caused by genetic mutations, confers a selective advantage that ultimately leads to invasive cancer 26. More importantly, the issuance of mutations is closely related to genomic stability at later stages. To better understand malignant tumor transformation, it is crucial to assess relevant indicators of genomic instability. The correlation of ARPC1A with TMB, MSI, and MMRs confirms its critical role in carcinogenesis.

The TME is a complex system consisting of immune cells, tumor-associated fibroblasts, endothelial cells, and ECM 27, 28. The composition of these components may differ depending on the tissue type and can co-evolve as the tumor progresses. In the TME, immune cells have a dual role. On one hand, they can act as immune surveillance by adopting an anti-tumor phenotype, which prevents tumor progression. On the other hand, they can adopt a pro-tumor phenotype under the influence of the TME, allowing tumors to escape or even supporting the TME, thus promoting tumor progression 29. In the present study, we observed a negative correlation between ARPC1A and B cells in almost all tumors. B cells have multiple functions in the TME as an essential immune system component. In most tumor studies, B cell infiltration has been associated with improved prognosis in cancer patients 30. However, a recent study has shown that B cells synthesize and secrete the neurotransmitter GABA, which induces IL-10 secretion from macrophages and suppresses anti-tumor T-cell responses 31. When evaluating immunomodulation, it was found that ARPC1A was strongly associated with immune, estimated, and stromal scores in different tumors. Additionally, it was observed that ARPC1A was negatively correlated with all immune stimulatory genes in THCA. However, in ACC and OV, ARPC1A was positively associated with both immune stimulatory and inhibitory genes. This phenomenon requires further in-depth study. In conclusion, the modulation of ARPC1A expression shows promise for shaping the immune microenvironment and discovering new anti-tumor therapeutic strategies.

The development of malignant tumors is characterized by high invasiveness and unrestricted proliferation 32. The activation of EMT is a critical process in the invasion and metastasis of cancer cells. During EMT, epithelial cells acquire the characteristics of mesenchymal cells, resulting in enhanced cell motility and migration 33, 34. Our GSEA in brain malignant gliomas and single-cell functional analysis revealed a positive correlation between ARPC1A and EMT, as well as critical signaling pathways for proliferation. *In vitro* experiments were performed using U87 and U251 cell lines. The results of CCK-8 with flow cytometry showed that ARPC1A knockdown suppressed the proliferative capacity of GBM cells. Furthermore, we found that ARPC1A knockdown inhibited the migration and invasion ability of GBM cells. Upon ARPC1A knockdown, E-cadherin expression increased, while N-cadherin and vimentin expression decreased. In various types of cancer, the deletion of E-cadherin is typically associated with increased expression of N-cadherin, which is considered essential for tumor cells to acquire invasiveness 35, 36. Moreover, overexpression of vimentin is associated with increased invasiveness and metastasis 37. Therefore, it can be concluded that ARPC1A plays a crucial role in regulating the malignant behavior of GBM cells, highlighting its potential as a therapeutic target.

Although TMZ is commonly used as a first-line therapeutic agent for GBM, over 50% of treatments fail due to drug resistance 38. GBM cells have robust DNA damage repair systems and complex repair mechanisms, which are essential in mediating resistance to TMZ. The present study concludes that ARPC1A increases the sensitivity of GBM to TMZ. Additionally, it was discovered that ARPC1A knockdown in TMZ-resistant cell lines reduced the expression of MGMT. MGMT is a crucial DNA damage repair gene whose expression is regulated by promoter methylation 39. When the promoter is hypomethylated, MGMT expression is increased. MGMT promoter methylation is a significant molecular marker for glioma patients and is linked to glioma prognosis and resistance to alkylating drugs like TMZ 40. These findings highlight the significant potential of ARPC1A in GBM therapy.

This study had some limitations. First, one must note that the results obtained from public data and statistical analyses may introduce heterogeneity or bias. Although these findings provide valuable guidance and information for further research, their direct clinical applicability is limited. Second, additional experiments are required to verify the relationship between ARPC1A expression and immune-related factors. Regarding ARPC1A expression and clinical data, we should obtain more patient samples and information to analyze the association between ARPC1A expression and clinical parameters of patients. Finally, this study was limited to *in vitro*, and it is necessary to include animal experiments with in-depth clinical studies to clarify that ARPC1A could be a therapeutic target for GBM.

## Conclusion

In conclusion, this study revealed that ARPC1A expression was upregulated in various tumors and correlates with poor prognosis. In addition, ARPC1A was significantly correlated with immune cells in TME, suggesting its potential as an immunotherapeutic target. The current study also found that ARPC1A acts as an oncogene in GBM and can modulate the effect of TMZ in treating GBM.

## Supplementary Material

Supplementary figures and tables.

## Abbreviations

ACC: adrenocortical carcinoma
ARP2/3: actin-related protein 2/3
ARPC1A: actin-related protein 2/3 complex subunit 1a
BLCA: bladder urothelial carcinoma
BRCA: breast invasive carcinoma
CESC: cervical squamous cell carcinoma and endocervical adenocarcinoma
CHOL: cholangiocarcinoma
COAD: colon adenocarcinoma
DFI: disease-free interval
DLBC: lymphoid neoplasm diffuse large B-cell lymphoma
DSS: disease-specific survival
ECM: extracellular matrix
EMT: epithelial-mesenchymal transition
ESCA: esophageal carcinoma
GBM: glioblastoma multiforme
GBMLGG: glioma
GDC: genomic data commons
GSEA: gene set enrichment analysis
GTEx: genotype-tissue expression
HNSC: head and neck squamous cell carcinoma
HPA: human protein atlas
KEGG: kyoto encyclopedia of genes and genomes
KICH: kidney chromophobe
KIRC: kidney renal clear cell carcinoma
KIRP: kidney renal papillary cell carcinoma
LAML: acute myeloid leukemia
LGG: brain lower grade glioma
LIHC: liver hepatocellular carcinoma
LUAD: lung adenocarcinoma
LUSC: lung squamous cell carcinoma
MESO: mesothelioma
MGMT: o6 -methylguanine-DNA methyltransferase
MMR: mismatch repair system
MSI: microsatellite instability
OS: overall survival
OV: ovarian serous cystadenocarcinoma
PAAD: pancreatic adenocarcinoma
PCPG: pheochromocytoma and paraganglioma
PFI: progression-free interval
PRAD: prostate adenocarcinoma
READ: rectum adenocarcinoma
SARC: sarcoma
SKCM: skin cutaneous melanoma
STAD: stomach adenocarcinoma
TCGA: the cancer genome atlas
TGCT: testicular germ cell tumors
THCA: thyroid carcinoma
THYM: thymoma
TISCH2: tumor immune single-cell hub 2
TMB: tumor mutation burden
TME: tumor microenvironment
TMZ: temozolomide
TNBC: triple negative breast cancer
UCEC: uterine corpus endometrial carcinoma
UCS: uterine carcinosarcoma
UVM: uveal melanoma
