# Supplementary material for: Experimental Validation and Multi-omics Analysis Identified ARPC1A as a Novel Oncogene and Potential Therapeutic Target in Glioblastoma
Source: J Cancer. 2024 May 30;15(12):3958–74. doi: 10.7150/jca.94552 (PMC11190758; doi:10.7150/jca.94552)
Supplement: Supplementary file 1 — Supplementary figures and tables. [file jcav15p3958s1.pdf]

Title: Experimental Validation and Multi-omics Analysis Identified ARPC1A As A Novel Oncogene And Potential Therapeutic Target In Glioblastoma

Journal: Journal of Cancer

Jun Yang1†; Chengcheng Xue2†; Zesong He1; Li Ying1; Wei Meng1; Meihua Li1; Na Zhang2*; Taohui Ouyang1*

1 Department of Neurosurgery, the 1st affiliated hospital, Jiangxi Medical College, Nanchang University, No.17, Yongwai Street, Nanchang, Jiangxi province, 330006, China

2 Department of Neurology, the 1st affiliated hospital, Jiangxi Medical College, Nanchang University, No.17, Yongwai Street, Nanchang, Jiangxi province, 330006, China

* Correspondence: ndyfy05017@ncu.edu.cn (TO); hustzhangna@163.com (NZ)

† These authors contributed equally to this work.

Table S1 The clinical parameters of the patients

**Characteristics**	**Number of cases**
Age (years)	
>60	3
<60	1
Gender	
Female	1
Male	3
Tumor size (Maximum diameter)	
≥ 5 cm	4
IDH status	
Wild-type	4

Note：IDH, isocitrate dehydrogenase

Table S2 Complete sequences of the three siRNAs

ARPC1A-1	forward: 5′-GAUUUCAUUCCAUUCUUGATT-3′, reverse: 5′-UCAAGAAUGGAAUGAAAUCTT-3′
ARPC1A-2	forward: 5′-GCUCCUAAGUGUGUCAUUUTT-3′, reverse: 5′-AAAUGACACACUUAGGAGCTT-3′
ARPC1A-3	forward: 5′-UGACAAUUUGGGAUUUCAATT-3′, reverse: 5′-UUGAAAUCCCAAAUUGUCATT-3′

Table S3 Antibody Information

Antibody name	Catalogue Number	Source of purchase
ARPC1A	#DF6215	Affinity Biosciences
E-cadherin	EM0502	Huabio
N-cadherin	EM1607-37	Huabio
Vimentin	MA5-11883	Thermo Fisher Scientific
GAPDH	#AF7021	Affinity Biosciences
MGMT	#AF5295	Affinity Biosciences

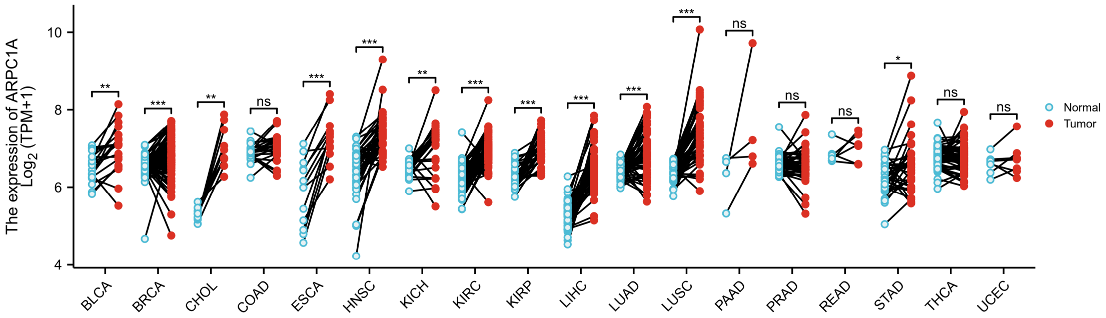

Figure S1 Differential expression of ARPC1A in paired tumor samples

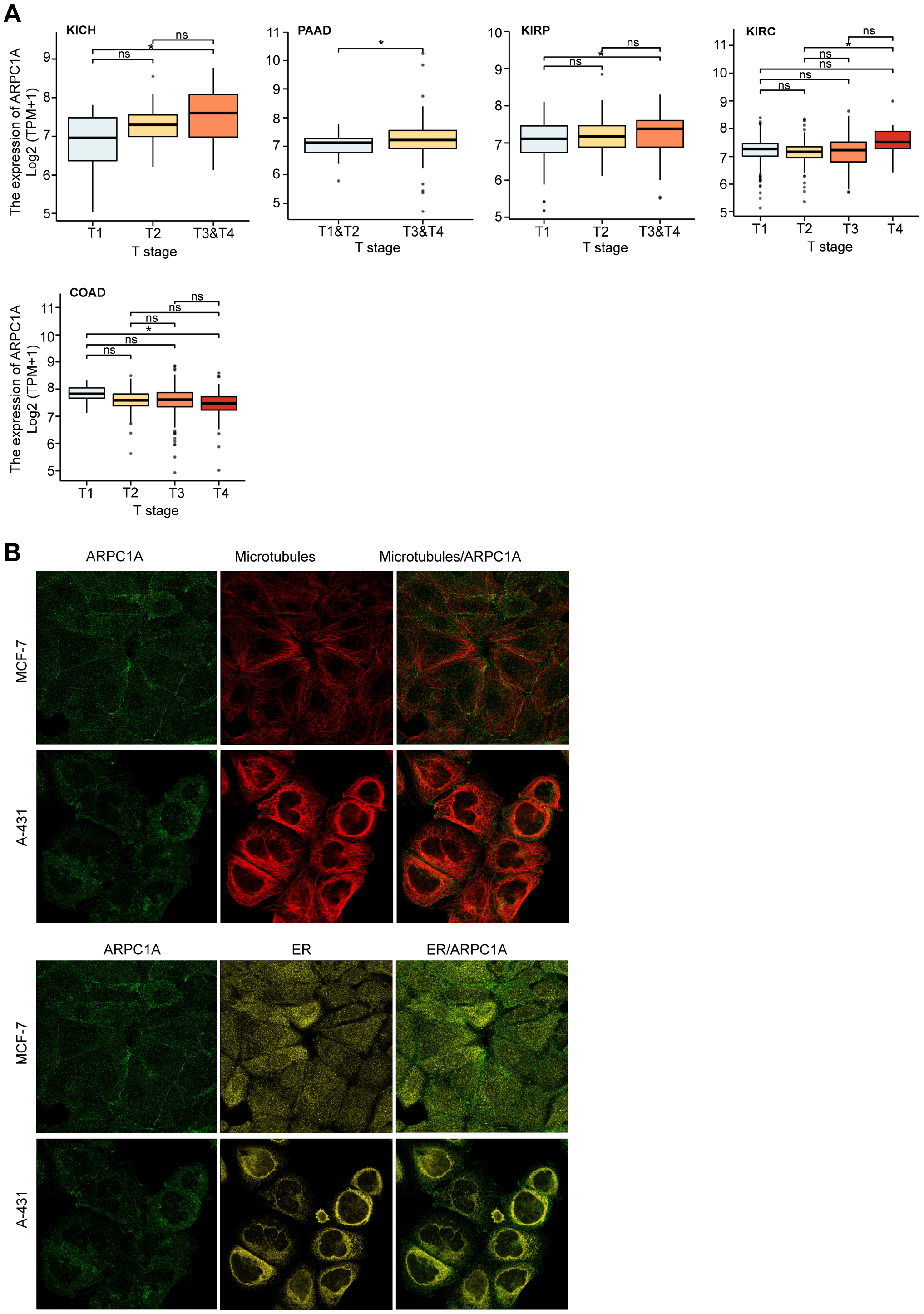

Figure S2: Expression pattern of ARPC1A in pan-cancer. **A** Correlation between ARPC1A expression and tumor stage. **B** Immunofluorescence images of ARPC1A in MCF-7 and A-431 cell lines were obtained from the HPA database

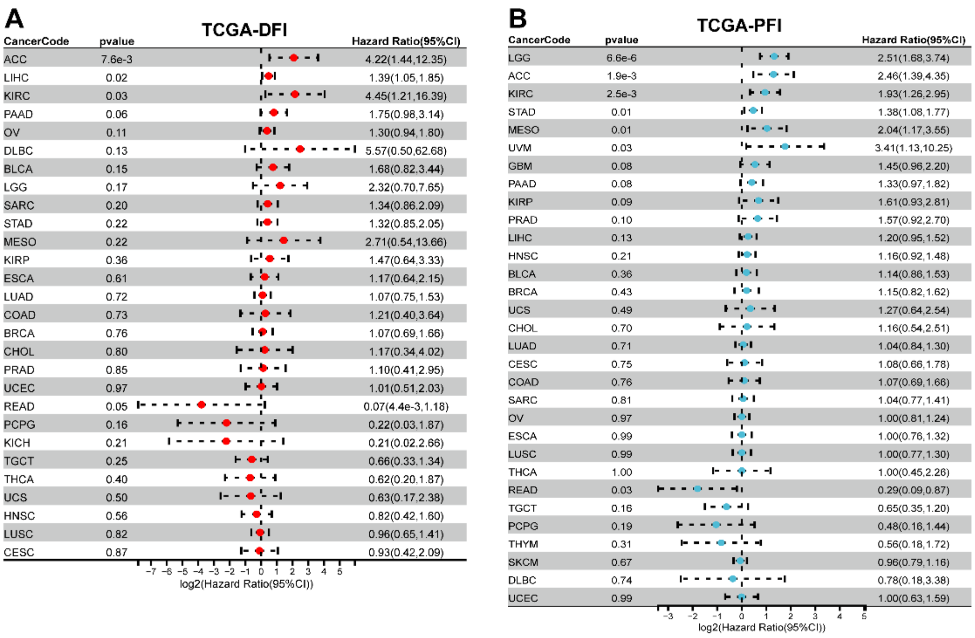

Figure S3 Prognostic value of ARPC1A in various tumors. **A-B** The forest plot showed the correlation of ARPC1A with DFI and PFI in tumors using the univariate Cox regression

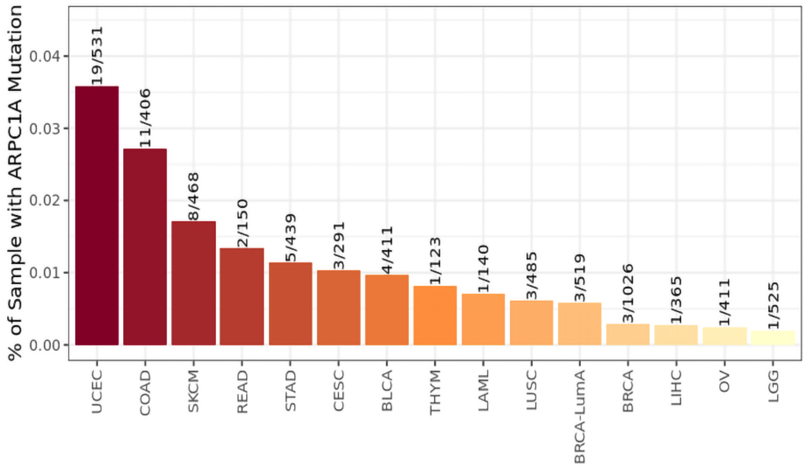

Figure S4 Mutation frequency of ARPC1A in multiple cancers
